# Barriers to Participation and Adherence in Cardiac Rehabilitation Programmes: A Scoping Review of Recent Evidence from Industrialized and Developing Countries

**DOI:** 10.31083/RCM45898

**Published:** 2026-03-12

**Authors:** Aliff Latir, Eliza Hafiz, Anwar Suhaimi

**Affiliations:** ^1^Department of Rehabilitation Medicine, Faculty of Medicine, Universiti Malaya, 50603 Kuala Lumpur, Malaysia; ^2^Centre for Physiotherapy Studies, Faculty of Health Sciences, Universiti Teknologi MARA (UiTM), Selangor Branch, Puncak Alam Campus, 42300 Bandar Puncak Alam, Selangor, Malaysia; ^3^Faculty of Sports and Exercise Science, Universiti Malaya, 50603 Kuala Lumpur, Malaysia

**Keywords:** cardiac rehabilitation, coronary artery disease, delivery of health care, industrialised countries, developing countries

## Abstract

Participation and adherence to cardiac rehabilitation (CR) remain low worldwide; meanwhile, differences in barriers between industrialized and developing countries have not been well synthesized. A scoping review was conducted following the Preferred Reporting Items for Systematic Reviews and Meta-Analyses extension for Scoping Reviews (PRISMA-ScR) guidelines to map recent evidence (2014–2025) on barriers to CR participation and adherence in industrialized and developing settings. Searches conducted in major databases identified 538 records, of which 19 met the inclusion criteria for thematic analysis. Participation in CR ranged from 12.3% to 81% in industrialized countries and from 5% to 70% in developing settings, while adherence ranged from 70.8% to 90.3% and from 20.4% to 71.3%, respectively. Reported barriers can be clustered into patient-level beliefs and perceptions, logistical and work-related constraints, comorbidities and health status, socioeconomic and demographic factors, psychological characteristics, and health-system and environmental limitations. A wide variation in CR utilization persists globally, with distinct patterns of barriers across industrialized and developing contexts. These findings highlight the need for setting-specific strategies to improve CR participation and adherence.

## 1. Introduction

Coronary artery disease (CAD) remains one of the leading causes of mortality and disability worldwide [1]. An estimated 126 million individuals—approximately 1.72% of the global population—are currently affected, and this burden continues to rise despite advancements in acute cardiac care [2]. Population ageing, persistent exposure to modifiable risk factors, and widening health disparities contribute to the escalating global impact of CAD, underscoring the urgent need for effective secondary prevention strategies [3, 4]. Cardiac rehabilitation (CR) is a cornerstone of such prevention, with robust evidence demonstrating reductions in recurrent cardiovascular events, hospital readmissions, morbidity, and mortality. Traditionally, CR is organised into sequential phases: Phase I (inpatient CR), delivered during the acute hospitalisation following a cardiac event, focuses on early mobilisation, clinical assessment, risk-factor education, and preparing patients for discharge; Phase II (outpatient CR), a medically supervised programme initiated shortly after discharge, emphasising structured exercise training, lifestyle and risk-factor modification, optimisation of cardioprotective therapies, and psychosocial support; and Phase III (maintenance), a long term maintenance programme guided by health professionals focusing on exercise, self-management, and lifestyle integration [5]. Contemporary CR has since expanded into a comprehensive, multidisciplinary framework that incorporates exercise training, lifestyle modification, psychosocial support, health education, therapeutic optimisation, and dietary and smoking-cessation counselling [6, 7]. Collectively, these components target key modifiable risk factors such as physical inactivity, obesity, and tobacco use [8].

Despite these benefits, CR utilisation remains suboptimal worldwide. Participation is frequently defined as attendance at the first outpatient CR session [9] or completion of at least one supervised session [10], while adherence refers to the proportion of enrolled patients who completed the prescribed programme (i.e., non-dropouts), with several studies classifying attendance of ≥75% of scheduled sessions as completion [11, 12]. Nonetheless, CR utilisation remains low despite the presence of standardised recommendations. Participation in many high-income countries ranged from 40% to 50% [13], while rates in low- and middle-income settings are even lower [14]. Among those who enrol, adherence is frequently inadequate, and lower completion has been associated with higher rehospitalisation rates and poorer long-term outcomes [15, 16].

Over the past two decades, research has gradually shifted from demonstrating the clinical efficacy of CR to investigating the complex, multifactorial barriers that limit patient engagement [17, 18]. These barriers span healthcare delivery factors, socioeconomic challenges, individual characteristics, and environmental constraints [19, 20]. Nevertheless, a critical gap remains in the literature. There is limited comparative evidence that examined differences in barriers to CR participation and adherence between industrialised and developing countries. Given the substantial structural and contextual discrepancies across health systems, such comparisons are essential for understanding the global variability in CR uptake and for informing context-appropriate strategies to improve access and adherence. The absence of this perspective restricts the applicability of existing evidence and hinders efforts to design scalable, equity-focused interventions.

Therefore, this scoping review aimed to (i) identify recent barriers to participation and adherence to CR among patients with CAD and (ii) compare these barriers between industrialised and developing countries. Understanding these differences will enable policymakers, healthcare providers, and stakeholders to prioritise actionable areas for intervention and to develop sustainable strategies to increase participation and adherence in CR, ultimately reducing the global burden of CAD.

## 2. Methodology

### 2.1 Study Design

Officially registered on Open Science Framework (https://osf.io/q4njd), this study utilised a scoping review approach. A scoping review is a flexible methodological tool for exploring rapidly evolving and emerging themes. The study design included a broader conceptual framework that explained several relevant study results. Five key methods proposed by Arksey and O’Malley [21] formed the basis of the scoping review framework: (1) Defining research questions, (2) Identifying relevant studies, (3) Selecting studies, (4) Data mapping, and (5) Compiling, summarising, and presenting the findings [22].

**Fig. 1.**
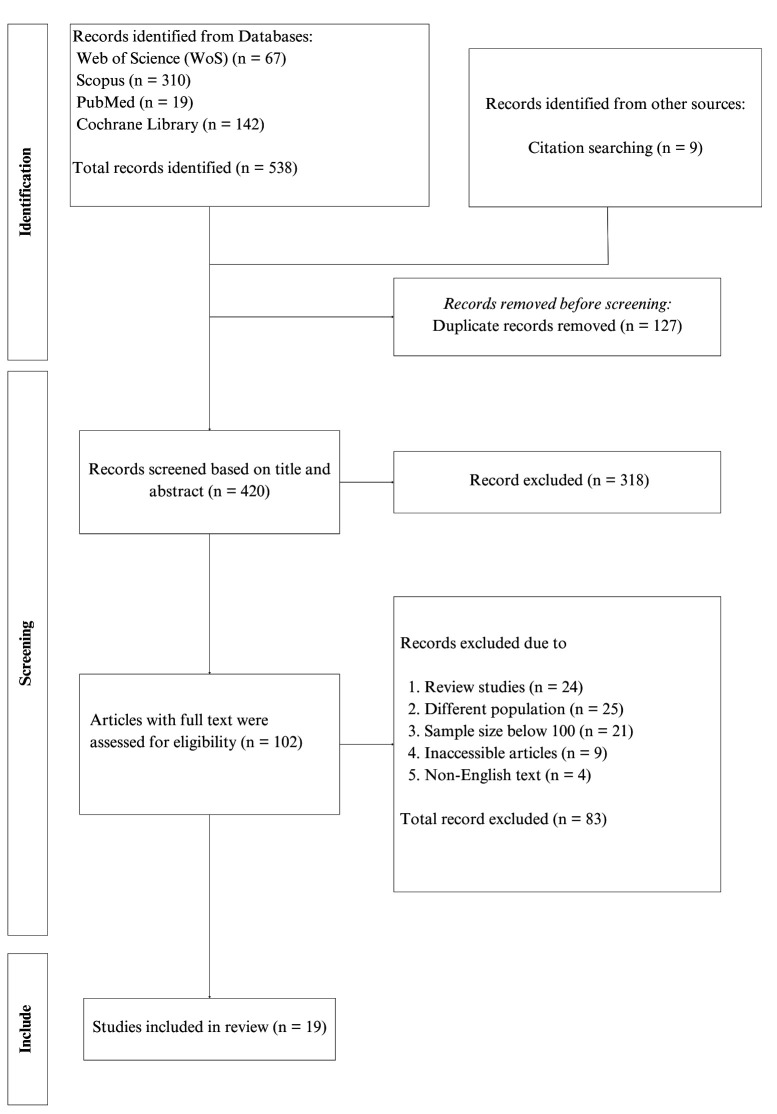
**PRISMA-ScR flow diagram illustrating the article selection process**. PRISMA-ScR, Preferred Reporting Items for Systematic Reviews and Meta-Analyses extension for Scoping Reviews.

The article selection process for this review was conducted using the Preferred Reporting Items for Systematic Reviews and Meta-Analyses extension for Scoping Reviews selection tool (see Fig. 1). The research question and selection criteria were developed using the Population, Concept, and Context framework, focusing on patients diagnosed with coronary artery disease who had undergone percutaneous coronary intervention, and examining barriers to participation and adherence in CR.

Articles meeting the inclusion criteria for this study include full-text English publications that used descriptive, cross-sectional, cohort, or mixed-method designs, focusing on barriers to participation and adherence. The stages of rehabilitation were also identified to identify various participation barriers and reduce study outcome bias. The search was limited to studies published between 2014 and 2025 to capture recent literature on CR barriers, and studies with a sample size of less than 100 were excluded.

### 2.2 Data Collection

A comprehensive search of studies exploring the barriers to participation and adherence was performed across four databases: Scopus, Cochrane Library, Web of Science (WoS), and PUBMED. A combination of specific keywords and Boolean operators was used in this step: coronary artery disease OR CAD OR coronary heart disease OR CHD OR myocardial infarction OR MI OR cardiovascular disease OR CVD AND barrier OR impediment OR obstacle AND participation OR adherence OR compliance OR engagement AND cardiac rehabilitation OR rehabilitation OR recovery.

Three independent reviewers confirmed that the selected article met the eligibility criteria. EndNote 21 software was used to identify duplicates during the initial selection phase. Subsequently, the reviewers assessed the titles, abstracts, and full texts for relevance to the research question, establishing inclusion and exclusion criteria before independently reviewing the filtered records. The articles retrieved met the following criteria: (i) studies on coronary patients referred by medical professionals and involved in CR, and (ii) studies exploring patients’ perceptions and beliefs, individual characteristics, hospital system-related factors, logistical factors, socioeconomic factors, and environmental barriers to CR participation and/or adherence among the study population. All three reviewers reached a consensus in cases of outcome discrepancies, and no disagreements arose regarding the suitability of the articles throughout the screening process.

An extraction table was established to characterise the findings related to the subject matter, which detailed the following information: (1) Study design, (2) Country, (3) Participants’ age (mean ± SD), (4) Participation and adherence rates, (5) Barriers, and (6) Phase and design of CR. Data analysis was conducted thematically, using a descriptive exploratory method adopted from Braun and Clark [23]. Firstly, the relevant data were identified and presented in tabular form, depending on the articles examined. All authors analysed and interpreted the data collected, focusing on barriers to CR in patients who had undergone PCI. The participation rates were classified into three groups: Low (≤50% or less), moderate (51–75%), and high (>75%). In this scoping review, the total number of patients attending at least one CR session was set as the minimum participation rate.

## 3. Results

### 3.1 Study Selection and Characteristics

The search across databases and **Supplementary Material** including grey literature, Google Scholar, and reference list screening yielded 538 articles. A total of 127 duplicates were omitted, followed by 318 articles after an initial review of titles and abstracts. After a detailed review of the remaining 102 full texts, an additional 83 articles were excluded. Finally, 19 articles fulfilled the criteria for data extraction and were included in this review [11, 12, 15, 24, 25, 26, 27, 28, 29, 30, 31, 32, 33, 34, 35, 36, 37, 38, 39]. Of the 19 studies, 11 were cross-sectional, three were either cohort or retrospective, and two used a mixed methods study design.

The shortlisted articles included studies from industrialised and developing countries. Eleven articles were published on industrialised nations: one each from Norway, Portugal, the United Kingdom, the Czech Republic, the USA, Singapore, Australia, and Spain; two from Korea; and one cross-national study covering eight European countries. Eight articles were from developing nations; four from China, one from Saudi Arabia, one from Brazil, and two from Malaysia. The combined total of study participants was 97,672, with an average age ranging from 55.8 to 71.0 years (see Table 1, Ref. [11, 12, 25, 26, 28, 29, 30, 31, 32, 33, 34, 35, 36, 37, 39], and Table 2, Ref. [11, 12, 15, 24, 27, 38]).

**Table 1.** **Characteristics of included studies examining barriers to CR participation**.

Authors	Design	Country	Sample size	Age, Mean (SD)	Findings		
					Participation rate (%)	Types/Phases of CR	Barriers
Almoghairi *et al*. [39]	Cross-sectional observational study	Saudi Arabia	104	59.5 (13)	36.4	Hospital-based/Phase I (Inpatient)	Distance from the healthcare facility
							Not informed about CR
							Lack of support from the clinician
							Not contacted by CR staff
Chong *et al*. [35]	Cross-sectional, correlational study	Malaysia	240	60.5 (10.6)	70	Hospital-based/Phase II (Outpatient)	Logistics
							Social support
							Co-morbidities/functional status
							Ethnicity
							Ability to drive
							Anxiety
Xie *et al*. [25]	Mixed-method study	China	160	N/A	24.4	Hospital-based/Phase II (Outpatient)	Distance from the healthcare facility
							Cost
							Time constraint
							Transportation
							Work responsibility
Winnige *et al*. [11]	Prospective observational study	Czech Republic	186	59.5 (8.8)	24.2	Hospital-based/Phase II (Outpatient)	Distance from the healthcare facility
							Work/time constraint
							Transportation
							Co-morbidities
Liu *et al*. [30]	Prospective observational study	China	380	67.0 (11.1)	5	Hospital-based/Phase II (Outpatient)	Distance from the healthcare facility
							Lack of awareness
							Weather
							Transportation
González-Salvado *et al*. [12]	Prospective observational study	Eight European countries	1633	≥65	68	Hospital-based/Phase I–II (Inpatient–Outpatient)	Employment status
							Living condition
Kim *et al*. [31]	Retrospective cohort study	Korea	64,982	N/A	1.5	Hospital-based/Phase II (Outpatient)	Age
							Gender (Female)
							Rural residence
							Low Charlson Comorbidity Index
Viana *et al*. [26]	Prospective cohort study	Portugal	939	63.5 (12.9)	Porto: 81	Hospital-based/Phases II and III (Outpatient–Maintenance)	Age
					NER: 66.7		Travel time
Chamosa *et al*. [36]	Retrospective study	Spain	756	58.0 (11.5)	79.1	Hospital-based/Phase II (Outpatient)	Age
							Living alone
							Distance from healthcare facility (>50 km)
							Medical history
Poh *et al*. [28]	Prospective observational study	Singapore	795	54.8 (9.7)	12.3	Hospital-based/Phase II (Outpatient)	Work
							Prefer self-exercise
							Time constraint
							Nationality (Foreigner)
Olsen *et al*. [29]	Observational cohort study	Norway	9013	63.1 (10.2)	27.6	Hospital-based/N/A	Gender
							Age
							Prior MI/CABG/ACS
							Educational level
							BMI
Foster *et al*. [34]	Cross-sectional study	United Kingdom	567	68.7 (10.5)	53	Hospital-based/N/A	Perceived need/healthcare factors
Khadanga *et al*. [33]	Prospective observational study	USA	294	68.3 (12)	60	N/A/Phase II (Outpatient)	Electronic referral
							Surgical diagnosis
							Smoking status
							Physician recommendation
							Educational level
							Social support
							EF status
							Physical function
Kim *et al*. [32]	Prospective study	Korea	173	63 (10)	46.2	Hospital-based/Phase II (Outpatient)	Symptom experience
							Socioeconomic factors
							Perceived susceptibility
							Perceived benefits
							Perceived severity
Chai *et al*. [37]	Prospective observational study	Malaysia	380	55.8 (10.3)	27.4	Hospital-based/Phase II (Outpatient)	Gender
							Race
							Travel time
							Ability to drive

Abbreviation: ACS, acute coronary syndrome; BMI, body mass index; CABG, coronary artery bypass graft surgery; EF, ejection fraction; MI, myocardial infarction; NER, northeastern region; N/A, not available; SD, standard deviation; CR, cardiac rehabilitation.

**Table 2.** **Characteristics of included studies examining barriers to CR adherence**.

Authors	Design	Country	Sample size	Age, Mean (SD)	Findings		
					Adherence rate (%)	Types/Phases of CR	Barriers
Beleigoli *et al*. [15]	Mixed-method study	Australia	16,159	71.0 (60–79)	75.4	N/A/N/A	Enrolled in a telephone-based programme
							Depression
							Living alone
							Diabetes
Santos *et al*. [27]	Cross-sectional observational study	Brazil	220	66.8 (11.6)	71.3	Hospital-based/Phase II (Outpatient)	Distance from the healthcare facility
							Cost
							Transportation
							Work/time constraint
Winnige *et al*. [11]	Prospective observational study	Czech Republic	186	59.5 (8.8)	70.8	Hospital-based/Phase II (Outpatient)	Travel
							Distance from the healthcare facility
							Work responsibilities
							Co-morbidities
González-Salvado *et al*. [12]	Prospective observational study	Eight European countries	1633	≥65	90.3	Hospital-based/Phase I–II (Inpatient–Outpatient)	Gender
							Co-morbidities
Cao *et al*. [38]	Prospective longitudinal study	China	300	60 (11.8)	64.66	Home-based/Phase II (Outpatient)	Age
							Perception of shared decision making
							Knowledge about risk factors
							Predisposing factors
							Under treatment
							Self management behaviour
							Life management
							Emotional management
Zhang *et al*. [24]	Retrospective study	China	391	59.89 (9.3)	20.38	Hospital-based/Phase II (Outpatient)	Support provided by the Life Club

Abbreviation: CR, cardiac rehabilitation; SD, standard deviation.

The CR designs in the reviewed articles varied, with most studies reporting hospital-based CR [11, 12, 24, 25, 26, 27, 28, 29, 30, 31, 32, 34, 35, 36, 37, 39] and one home-based programme [38]. Meanwhile, two studies did not specify the rehabilitation design [15, 33]. Most included studies were conducted in Phase II outpatient CR, where 13 evaluated barriers or participation [11, 24, 25, 27, 28, 30, 31, 32, 33, 35, 36, 37, 38]. Only one study examined Phase I inpatient CR [39]. Several studies also spanned multiple phases; one covering Phases I–II and another Phases II–III, reflecting mixed designs or longitudinal programme structures [12, 26]. Three studies did not specify the CR phase [15, 29, 34].

The CR participation ranged from 12.3% to 81% in industrialised countries and 5% to 70% in developing settings. Adherence in industrialised regions was between 70.8% and 90.3%, while in developing countries reported to be 20.4% to 71.3%. Across the included studies, 11 reported low participation [11, 15, 24, 25, 28, 29, 30, 31, 32, 37, 39], four reported moderate participation [12, 33, 34, 35], and two described moderate-to-high rates [26, 36]. Meanwhile, four studies reported low rates of adherence [11, 24, 27, 38], whereas the remaining two reported high rates [12, 15].

### 3.2 Barriers to CR Participation and Adherence: Industrialized Versus Developing Countries

**Fig. 2.**
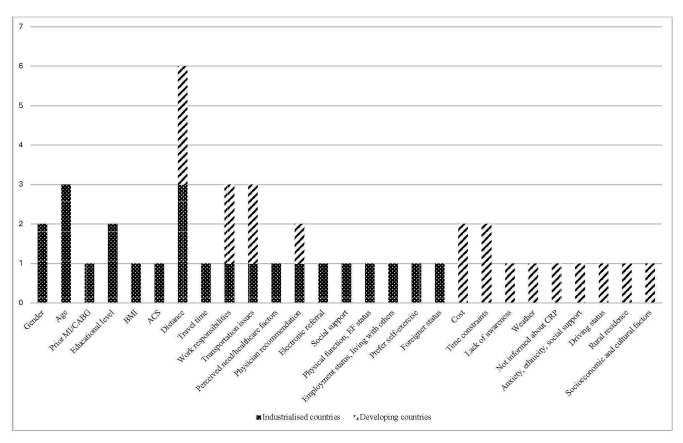
**Barriers to participation in CR: industrialised vs. developing countries**. CR, cardiac rehabilitation.

Fig. 2 illustrates the barriers to participation in industrialised and developing countries. Gender, age, and educational level were the most commonly reported barriers in industrialised countries. In contrast, studies in developing countries reported higher barriers related to CR costs and time constraints. Distance to CR centres, work responsibilities, and transportation issues were frequently reported as barriers in both settings, although these factors were predominant in developing countries. Additionally, a lack of clinician recommendation was a common barrier for CR, with similar prevalence in both settings.

**Fig. 3.**
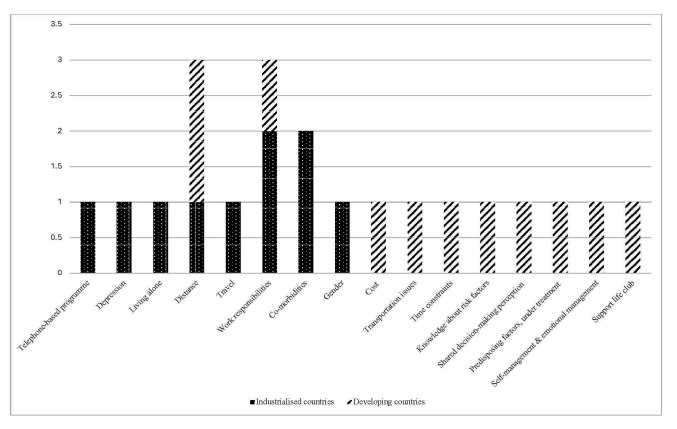
**Barriers to adherence in CR: industrialised vs. developing countries**. CR, cardiac rehabilitation.

Fig. 3 compares barriers to CR adherence between the two settings. The most common barriers in industrialised countries included work responsibilities and comorbidities. Distance to CR centres and work responsibilities were widespread barriers in both settings, particularly in developing countries.

### 3.3 Barriers Affecting CR Participation and Adherence

#### 3.3.1 Patient’s Perception and Belief

Table 3 (Ref. [11, 12, 15, 24, 25, 26, 27, 28, 29, 30, 31, 32, 33, 34, 35, 36, 37, 38, 39]) summarises the thematic patterns of barriers affecting cardiac rehabilitation participation and adherence across the included studies, integrating key quantitative findings with their contextual interpretations. Two studies examined the link between perceived needs and CR participation [34, 35]. Foster *et al*. [34] identified a lack of perceived need as a key barrier to the programme, leading to a 50% drop in attendance (odds ratio (OR) = 0.02, [confidence interval (95% CI): 0.01–0.06]). Similarly, Chong *et al*. [35] found that lower perceived need scores were associated with a reduced likelihood of participation [adjusted odds ratio (AOR) = 0.18, (95% CI: 0.09–0.35)]. Meanwhile, Kim *et al*. [40] revealed that higher levels of perceived benefits [OR = 1.09, (95% CI: 1.02–1.17)], perceived severity [OR = 1.04, (95% CI: 1.00–1.08)], and perceived susceptibility [OR = 1.22, (95% CI: 1.08–1.39)], positively correlated with increased CR attendance. A greater perception of shared decision making was strongly associated with higher adherence to exercise-based home CR [Exp (B) = 1.14, (95% CI: 1.02–1.28)], as reported by Cao *et al*. [38]. Finally, 20.1% of patients declined participation in exercise-based CR because of personal preferences for self-exercise, as stated by Poh *et al*. [28].

**Table 3.** **Thematic summary of barriers affecting CR participation and adherence**.

Author/year	Themes	Results	Interpretation of findings
Patient perceptions and beliefs			
Chong *et al*., 2024 [35]	Perceived needs/benefits/severity/susceptibility	Perceived needs (AOR = 0.18, 95% CI: 0.09–0.35)	Patients with lower scores in perceived needs were associated with decreased odds of participation
Cao *et al*., 2021 [38]		Perception shared decision making (Exp (B) = 1.14; 95% CI: 1.02–1.28)	Shared decision-making was identified as a factor that influenced adherence to home exercise-based CR
Kim *et al*., 2021 [32]		Perceived benefits (OR = 1.09; 95% CI: 1.02–1.17)	Patients with higher scores in perceived benefits had higher odds of attendance
		Perceived severity (OR = 1.04; 95% CI: 1.00–1.08)	Patients with higher scores in perceived severity had higher odds of attendance
		Perceived susceptibility (OR = 1.22; 95% CI: 1.08–1.39)	Patients with higher scores in perceived susceptibility had higher odds of attendance
Foster *et al*., 2021 [34]		Lack of perceived needs (OR = 0.02; 95% CI: 0.01–0.06)	Lack of perceived need for CR was the sole significant factor associated with a 50-fold reduction in attendance
Poh *et al*., 2015 [28]		Preference for self-exercise (20.1%)	Preference for self-exercise is one of the reasons patients refused to participate in exercise-based CR
Healthcare system			
Khadanga *et al*., 2021 [33]	Healthcare providers’ support	Physician support (OR = 1.68; 95% CI: 1.34–2.11)	A stronger physician recommendation was associated with participation
Almoghairi *et al*., 2024 [39]		Not informed about CR (78%)	An estimated 78% non-participating patients reported not being informed about CR by their healthcare providers
Almoghairi *et al*., 2024 [39]	Healthcare providers’ support	Lack of support from the clinician (88%)	An estimated 88% of non-participating patients considered the lack of clinician support to be a significant barrier
		Not contacted by CR staff (91%)	Approximately 91% of non-participating patients cited the lack of communication with CR staff as a major barrier to their participation
Beleigoli *et al*., 2024 [15]	Program referral and delivery	Enrolled on a telephone-based programme (OR = 0.26; 95% CI: 0.18–0.38)	Enrolment via the telephone programme was linked to a higher rate of CR non-completion as opposed to the face-to-face programme
Khadanga *et al*., 2021 [33]		Electronic referral (OR = 8.79; 95% CI: 4.18–18.45)	Electronic referral usage was an independent predictor of participation
Logistical barriers			
Santos *et al*., 2023 [27]	Distance	Distance to centre, PHA versus PLA (CRBS 1.0 vs. 1.6), *p* = 0.001	PLAs demonstrated a greater distance barrier compared to PHAs
Winnige *et al*., 2021 [11]		Distance to centre (CRBS = 3.08)	Distance from the centre was rated the most significant barrier to low participation and adherence
Almoghairi *et al*., 2024 [39]		Hospital-based CR is too far from home (69%)	Most patients considered hospital-based CR located far from their homes as a barrier to participation
Xie *et al*., 2022 [25]		Distance to centre (CRBS = 3.29 ± 1.57)	Driving distances of more than 40 minutes were deemed too far for participants
Liu *et al*., 2021 [30]	Distance	Non-participant vs. participant (3.7 ± 0.9 vs. 2.8 ± 1.2), *p* = 0.013	Non-participants perceived a greater distance as a barrier as compared to participants
Chong *et al*., 2024 [35]		Logistical factors (OR = 0.22; 95% CI: 0.12–0.38)	Higher scores in logistical factors were associated with decreased odds of participation
Viana *et al*., 2018 [26]		Distance from home to centre (84.6% of NER, Portugal)	Limited centres and requiring patients to travel far distances to participate in the programme were major reasons for non-participation
Chamosa *et al*., 2015 [36]		Living further than 50 km from the centre (OR = 2.90; 95% CI: 1.29–6.41)	Living beyond 50 km from the CR centre increased the risk of non-enrollment by threefold
Chai *et al*., 2019 [37]		Travel time (*p* = 0.00)	CR enrollers mostly travelled less than an hour from home to the hospital
Kim *et al*., 2020 [31]		Rural resident (OR = 1.38; 95% CI: 1.06–1.79)	Participation from rural residents was lower than that in urban areas
Chong *et al*., 2024 [35]	Transportation	Driving status (OR = 0.29; 95% CI: 0.13–0.63)	Participants who did not drive to the CR centre were more likely to participate in the programme
Chai *et al*., 2019 [37]		Able to drive (*p* = 0.009)	Patients who enrolled in CR were more likely to be able to drive
Santos *et al*., 2023 [27]		PHA vs. PLA (1.38 vs. 1.10), *p* = 0.005	PHAs demonstrated greater transportation barriers than PLAs
Xie *et al*., 2022 [25]		Inconvenient traffic (CRBS = 2.99 ± 1.50)	Living in rural areas without public transport acted as a barrier to participation
Liu *et al*., 2021 [30]		Non-participant vs. participant (CRBS = 3.5 ± 1.0 vs. 2.6 ± 1.1)	Transportation issues were one of the main barriers perceived by the non-participants
Winnige *et al*., 2021 [11]	Transportation	Transportation (CRBS = 2.17)	Transportation issues were rated as one of the significant barriers to participation.
Work or time conflicts			
Winnige *et al*., 2021 [11]	Work or time conflicts	Lack of time (CRBS = 2.37 ± 1.55)	Lack of time is a significant barrier to CR enrolment
		Work responsibilities (CRBS = 2.78 ± 1.39)	Work responsibilities were significant barriers to CR enrolment and adherence
Poh *et al*., 2015 [28]		Busy work schedule (37.5%)	A busy work schedule was a common reason for patients to decline participating in CR
Santos *et al*., 2023 [27]		Time constraint, PLAs vs. PHAs (1.39 vs. 1.00), *p* = 0.02	PLAs perceived time constraint as a barrier to adherence more than PHAs
Xie *et al*., 2022 [25]		Time constraint (CRBS = 2.48 ± 1.49)	Time constraint was a major barrier to CR participation
Comorbidities and health status			
Cao *et al*., 2021 [38]	Comorbidities	Risk factors (Exp (B) = 10.9, 95% CI: 2.7–43.7)	Presence of risk factors was a key factor influencing adherence to home exercise-based CR
Beleigoli *et al*., 2024 [15]		Diabetes (OR = 1.48, 95% CI: 1.0–2.1)	Having diabetes was associated with CR non-completion
Winnige *et al*., 2021 [11]		Co-morbidities (CRBS = 2.13 ± 1.33)	Comorbidities were significant barriers to CR enrolment and adherence
Chong *et al*., 2024 [35]		Co-morbidities (AOR = 8.2, 95% CI: 3.8–17.8)	Higher scores in comorbidities/functional status were associated with increased odds of CR participation
González-Salvado *et al*., 2021 [12]		Co-morbidities (RA, heart valve disease, COPD, nephropathy), *p * < 0.05	Some comorbidity features, such as RA, heart valve disease, COPD, and nephropathy, were associated with CR dropouts
Socioeconomic and demographic factors			
Chong *et al*., 2024 [35]	Ethnicity	Chinese (OR = 0.19; 95% CI: 0.08–0.49), Indian (OR = 0.49; 95% CI: 0.2–1.2)	Chinese and Indian patients were less likely to participate in CR compared to Malays
Chai *et al*., 2019 [37]		Malay (*p* = 0.00)	Patients who enrolled in CR were mostly Malays
Xie *et al*., 2022 [25]	Cost	CR cost (CRBS = 2.76 ± 1.43)	High cost was a significant barrier to CR enrolment
Santos *et al*., 2023 [27]		CR cost, PLA versus HLA (1.10 vs. 1.00), *p* = 0.048	PLAs attending private CR perceived greater financial barriers than PHAs
Kim *et al*., 2021 [32]	Social status	Higher perceived socioeconomic status (OR = 2.9; 95% CI:1.3–6.6)	Higher perceived socioeconomic status was a significant predictor of CR attendance
Olsen *et al*., 2018 [29]		Attained level of education (OR = 1.5; 95% CI: 1.3–1.7)	Patients with a higher educational level were more likely to participate in CR
Khadanga *et al*., 2021 [33]		Higher educational attainment (OR = 1.71; 95% CI: 1.07–2.75)	Higher educational attainment was favourably associated with CR participation
González-Salvado *et al*., 2021 [12]		Larger proportion of employed non-participant (*p* = 0.02)	There was a significant difference in employment status between employed non-participants vs. participants in Zwolle, Netherlands
Individual and psychological characteristics			
Kim *et al*., 2020 [31]	Age	40–60 years (OR = 2.8; 95% CI: 2.3–3.5), 61–70 years (OR = 2.3; 95% CI: 1.8–2.8)	The younger age group demonstrated greater odds of CR participation than the older age group
Chamosa *et al*., 2015 [36]		Age (OR = 1.1; 95% CI: 1.0–1.1)	Non-participants were older than the participants
Olsen *et al*., 2018 [29]		60–69 years (OR = 0.6; 95% CI: 0.6–0.7), >70 years (OR = 0.3; 95% CI: 0.3–0.4)	The odds of attending CR decreased with increasing age
Viana *et al*., 2018 [26]	Age	Porto (OR 0.95; 95% CI 0.93–0.97 per year of age)	The probability of referral to CR decreased with age in both regions
		NER (OR 0.95; 95% CI 0.91–0.98 per year of age)	
Cao *et al*., 2021 [38]		Age (Exp (B) = 0.77; 95% CI: 0.69–0.87)	Age was the primary factor that affected adherence to home- and exercise-based CR in patients post-PCI
Kim *et al*., 2020 [31]	Gender	Male (OR = 2.0; 95% CI: 1.7–2.5)	More male patients participated in CR than their female counterparts
Chai *et al*., 2019 [37]		Male (*p* = 0.01)	Patients who enrolled in CR were more likely to be male
Olsen *et al*., 2018 [29]		Male (AOR = 0.7, 95% CI: 0.6–0.8)	Males had a lower probability of participating in CR compared to females
González-Salvado *et al*., 2021 [12]		Female, (Santiago, *p* = 0.01; Bern, *p* = 0.03)	Females were significantly more likely to leave the programme than males in Santiago
Beleigoli *et al*., 2024 [15]	Mental health	Depression (OR = 1.54; 95% CI: 1.1–2.0)	Depression at baseline was associated with CR non-completion
Cao *et al*., 2021 [38]		Emotional management (Exp (B) = 1.3; 95% CI: 1.0–1.7)	Emotional management impacted adherence to home exercise-based CR
Chong *et al*., 2024 [35]		Anxiety (AOR = 3.7; 95% CI: 1.2–11.1)	A higher level of perceived anxiety was associated with increased odds of CR participation
Environmental factors			
Chong *et al*., 2024 [35]	Social support	Perceived social support (AOR = 1.38; 95% CI: 1.00–1.90; *p* = 0.048)	Higher levels of perceived social support from friends were associated with increased odds of CR participation
Zhang *et al*., 2022 [24]		Support life club (OR = 27.38; 95% CI 10.2–73.6) (*p * < 0.000)	Patients having Life Club support attended more CR sessions than the control group
Chamosa *et al*., 2015 [36]		Living alone (OR = 4.54; 95% CI: 2.53–8.16)	Living alone was associated with lower CR participation
González-Salvado *et al*., 2021 [12]		Living with others (Santiago and Zwolle, *p * < 0.001, respectively).	Patients in Santiago and Zwolle who were living with others were significantly more likely to participate in CR programmes
Beleigoli *et al*., 2024 [15]		Living alone (OR = 1.38; 95% CI: 1.0–1.9)	Living alone was associated with CR non-completion
Khadanga *et al*., 2021 [33]		Social support (OR = 1.01; 95% CI: 1.00–1.12)	Social support is favourably associated with CR participation
Liu *et al*., 2021 [30]	Weather	Severe weather, non-participant vs. participant (3.5 ± 0.9 vs. 2.7 ± 1.3) *p* = 0.013	Severe weather was the primary barrier reported by the non-participant

Abbreviation: AOR, adjusted odd ratio; CI, confidence interval; CR, cardiac rehabilitation; CRBS, cardiac rehabilitation barriers score; COPD, chronic obstructive pulmonary disease; NER, northeastern region; OR, odd ratio; PCI, percutaneous coronary intervention; PHA, high adherence patients; PLA, low adherence patients; RA, rheumatoid arthritis.

#### 3.3.2 Healthcare System

Two studies identified physicians as a key facilitator for CR participation [33, 39]. Effective physician recommendations were linked to a significant increase in CR uptake [OR = 1.68, (95% CI: 1.34–2.11)] [33], whereas lack of clinician support, insufficient information about CR, and poor communication from staff contributed to non-participation [39]. Telephone-based programmes were the least successful and associated with lower completion rates [OR = 0.26, (95% CI: 0.18–0.38)] [15], while electronic referrals significantly improved patient participation in CR rehabilitation [OR = 8.79, (95% CI: 4.18–18.45)] [33].

#### 3.3.3 Logistical Barriers

Several studies discovered an inverse relationship between greater distance to CR centres and participation rates. Living more than 50 km from a CR centre was linked to almost triple the odds of non-participation [OR = 2.90, (95% CI: 1.29–6.41)] [36]. Kim *et al*. [31] observed that rural residents had lower participation rates compared to urban residents [OR = 1.38, (95% CI: 1.06–1.79)]. In rural areas of Eastern Malaysia, CR enrollees typically travelled less than an hour to the centres [37]. Distance also impacted CR programmes in China, as evidenced by higher perceived barriers reported by non-participants [25, 30]. Likewise, most respondents in Saudi Arabia (69%) and the Northeastern region of Portugal (84.6%) cited distance as the main reason for non-participation in CR [26, 39]. Meanwhile, two studies emphasised the impact of distance on adherence. Santos *et al*. [27] discovered that low adherence patients reported significantly distance-related obstacles than high adherence patients (CRBS score:1.6 vs. 1.0), while Winnige *et al*. [11] confirmed that distance was a major barrier to adherence (CRBS score: 3.08).

Transportation is another major hurdle to CR participation. A study by Chong *et al*. [35] revealed that higher scores in logistical factors were associated with lower participation rates [OR = 0.22, (95% CI: 0.12–0.38)], and individuals who did not drive recorded significantly higher participation rates [OR = 0.29, (95% CI: 0.13–0.63)]. In contrast, those who could drive were more likely to participate in a study by Chai *et al*. [37]. Inconvenient traffic conditions in rural areas were also a major barrier to participation (CRBS score: 2.99 and 2.17, respectively) [11, 25]. Liu *et al*. [30] and Santos *et al*. [27] confirmed these findings, reporting that transportation was a key barrier to participation and adherence (CRBS score: 3.5 and 1.38, respectively).

#### 3.3.4 Work or Time Conflicts

Multiple studies have emphasised the influence of time constraints on CR. Two studies indicated that time scarcity was a significant barrier (CRBS: 2.37 and 2.48, respectively) [11, 25]. Santos *et al*. [27] demonstrated that perceived time constraints were higher among low adherence patients than their high adherence counterparts (CRBS scores: 1.39 vs 1.00). Work commitments were also highlighted as a major obstacle to CR participation. Winnige *et al*. [11] stated that work responsibilities received high barrier ratings for CR participation and adherence (CRBS scores: 2.37 and 2.78, respectively). Similarly, Poh *et al*. [28] reported that 37.5% of patients who declined CR cited busy work schedules as the primary non-participating reason.

#### 3.3.5 Comorbidities and Health Status

Chong *et al*. [35] indicated that patients with higher comorbidities or better functional status scores were more likely to participate in CR [AOR = 8.2, (95% CI: 3.8–17.8)]. On the contrary, Winnige *et al*. [11] found that comorbidities pose significant challenges to patient participation and adherence (CRBS score: 2.13). Meanwhile, Beleigoli *et al*. [15] reported that patients with diabetes were more likely to discontinue their CR programme [OR = 1.48, (95% CI: 1.0–2.1)], and Gonzalez-Salvado *et al*. [12] attributed higher dropout rates to specific comorbidities (nephropathy, valvular heart disease, chronic obstructive pulmonary disease, and rheumatoid arthritis). Additionally, the presence of risk factors was identified as a significant predictor of poor adherence to home-based CR [Exp (B) = 10.9, (95% CI: 2.7–43.7)] [38].

#### 3.3.6 Socioeconomic and Demographic Factors

Two studies highlighted the impact of ethnic differences on CR participation in a multiracial country. Chong *et al*. [35] reported that individuals of Chinese (OR = 0.19, [95% CI: 0.08–0.49]) and Indian (OR = 0.49, [95% CI: 0.2–1.2]) descent were less likely to engage in CR compared to Malays. This finding is supported by Chai *et al*. [37], who observed that most CR participants were Malays (*p* = 0.00). Furthermore, financial barriers emerged as key factors for CR participation. High cost of CR was a significant obstacle (CRBS score: 2.76) [25], where low adherence patients perceived a greater financial burden than their high adherence counterparts (CRBS scores: 1.10 vs. 1.00) [27]. Other factors that influence CR participation were socioeconomic status and educational attainment. Kim *et al*. [31] reported that a higher perceived socioeconomic status was a strong predictor of CR participation (OR = 2.9, [95% CI: 1.3–6.6]). Similarly, higher educational attainment was significantly associated with a greater likelihood of CR participation [29, 33]. Nevertheless, employed individuals were significantly more likely to be non-participants than unemployed individuals [12].

#### 3.3.7 Individual and Psychological Characteristics

Studies have consistently shown that age significantly impacts CR participation and adherence. Individuals between the ages of 40 and 60 had the highest likelihood of participating compared to older age groups (OR = 2.8, [95% CI: 2.3–3.5]) [31]. Other studies also discovered that non-participants tended to be older, with those over 70 showing markedly lower odds of participation [29, 36]. This trend was further supported by Viana *et al*. [26], who reported a lower likelihood of CR referral with each additional year of age in the Porto district and the Northeastern region of Portugal. Additionally, Cao *et al*. [38] noted that age negatively affected adherence to exercise-based CR [Exp (B) = 0.77, (95% CI: 0.69–0.87)].

Reports regarding gender and CR participation varied. Two studies reported higher participation rates among males [OR = 2.0, (95% CI: 1.7–2.5); *p* = 0.01, respectively] [31, 37]. González-Salvado *et al*. [12] also observed that females were more likely to drop out of CR in specific locations, such as Santiago (*p* = 0.01) and Bern (*p* = 0.03). Conversely, Olsen *et al*. [29] discovered that males were less likely to participate than females [AOR = 0.7, (95% CI: 0.6–0.8)].

Mental health factors were linked to CR participation. Beleigoli *et al*. [15] highlighted the association between depression and low programme adherence [OR = 1.54, (95% CI: 1.1–2.0)]. Likewise, Cao *et al*. [38] demonstrated that adherence improved with better emotional management (Exp (B) = 1.3, [95% CI: 1.0–1.7]). On the contrary, higher anxiety levels were a significant predictor that increased the odds of CR participation (AOR = 3.7, [95% CI: 1.2–11.1]), according to Chong *et al*. [35].

#### 3.3.8 Environmental Factors

Perceived social support has been linked to CR participation in several studies. For instance, Chong *et al*. [35] stated that individuals receiving higher levels of peer support were more inclined to participate in CR [AOR = 1.38, (95% CI: 1.00–1.90)]. Similarly, Khadanga *et al*. [33] found a positive association between general social support and CR engagement [OR = 1.01, (95% CI: 1.00–1.12)], while González-Salvado *et al*. [12] observed significantly higher participation rates among those living with others in Santiago (Spain) and Zwolle (The Netherlands) (*p* < 0.0010). People living alone were also less likely to participate in CR (OR = 4.54, [95% CI: 2.53–8.16]) [36]. Meanwhile, individuals who received support from the Life Club were significantly more likely to adhere to and complete the programmes than those who did not (OR = 27.38, [95% CI: 10.2–73.6]) [24]. Beleigoli *et al*. [15] supported this finding by reporting that individuals living alone had an increased likelihood of CR non-completion [OR = 1.38, (95% CI: 1.0–1.9)].

## 4. Discussion

Cardiac rehabilitation is fundamentally a multidisciplinary programme that integrates health education, psychosocial support, lifestyle counselling, and therapeutic optimisation. In this review, several barriers to CR were identified, including inadequate knowledge of the disease and the associated risk factors [30, 38], misaligned perceptions of needs and benefits [32, 34], and insufficient social support [15]. These findings reflected the areas for improvement across the broader CR pathway, instead of issues solely linked to exercise.

A study by Conte *et al*. [41] demonstrated that most patients began CR with limited awareness of key modifiable cardiovascular risk factors, often underestimating the significance of physical inactivity, smoking, and diabetes. These gaps can be addressed by strengthening the educational, psychosocial, and lifestyle components of CR to enhance patient understanding, build confidence, and support sustained engagement. For instance, personalised training plans and the integration of technological tools have been proposed to educate CR participants to improve their awareness and adherence. While supervised exercise mitigates adverse cardiac remodelling and restores physical function, comprehensive education on cardiovascular risk factors, therapies, lifestyle modification, and therapeutic optimisation is equally critical for reducing morbidity and mortality [7].

Beyond individual-level influences, the broader distribution of barriers across contexts reflects deeper systemic shortcomings that continue to undermine equitable access to CR. In industrialised countries, persistently lower participation among older adults, women, and individuals with limited education signals entrenched inequities that current programme designs have yet to address effectively. Meanwhile, the dominance of financial, geographical, and time-related barriers in developing countries indicated structural deficiencies in health system planning, resource allocation, and service integration.

Logistical challenges, such as long travel distances, unreliable transportation, and inflexible work demands, also impacted CR participation and highlighted infrastructural gaps that remained unresolved despite existing evidence in the literature. The convergence of participation and adherence barriers further suggested that attrition stems not simply from individual motivation, but from a complex interplay of clinical, socioeconomic, and systemic constraints. Collectively, these findings underscore the need for CR programmes to move beyond traditional exercise-centric approaches and adopt context-responsive strategies that address social determinants and structural barriers to sustained participation and adherence.

Home-based CR delivers training, education, and lifestyle counselling remotely, often relying on digital tools such as wearables, mobile applications, and telemonitoring systems. Within this model, 64.66% of patients demonstrated good adherence, but was slightly lower than previously reported rates (73%–95%) [42, 43]. This change suggested that the unique demands of home-based delivery may amplify some barriers not typically encountered in hospital-based programmes. Generally, participants of home-based CR are required to self-regulate without real-time oversight, rendering behavioural determinants such as motivation, self-efficacy, and perceived social support to become disproportionately influential [44]. Likewise, the digital infrastructure underpinning home-based CR means that adherence is highly sensitive to technological factors, including platform usability, digital literacy, and access to reliable devices [45]. In contrast, centre-based CR are packaged with structured schedules, supervised sessions, and immediate professional feedback. Despite contributing to sustained participant engagement, this traditional approach has barriers observed in different phases. In Phase I (inpatient), challenges are commonly related to early referral, inconsistent delivery of inpatient education, and gaps in communication [46]. Patients often reported being uninformed about CR, lacking clinician support, or not being contacted by the CR staff [39]. Failures in continuity contribute to weak enrolment into Phase II (outpatient) services, despite the high participation during hospitalisation. Once patients transition to Phase II, the dominant barriers shift toward logistical issues such as distance, transportation, time constraints, and work responsibilities, as reflected across many outpatient studies [15, 25, 28, 33, 35, 39].

Patient perceptions, beliefs, and preferences were recognised as key factors influencing participation and adherence to the CR programme. Studies have shown that CR participation was positively affected by perceived needs, benefits, severity, and vulnerability [32, 34, 35]. These findings align with earlier studies, where increased perceived vulnerability and severity prompted changes in health behaviour, such as routine health screenings, treatment adherence, and avoiding additional injury [47]. Perceived vulnerability and severity were also combined in several studies and were identified as perceived threats that motivated behavioural changes and responsibility for patients’ health [48, 49]. Additionally, collaborative approaches such as shared decision making potentially improved CR adherence [38]. In summary, recognising individuals’ perceptual factors and fostering shared decision-making may enhance CR engagement and efficacy.

Physician support and programme delivery design emerged as critical healthcare system–level determinants of CR participation and adherence. Several studies demonstrated that strong physician recommendations, comprehensive patient education, and consistent follow-up substantially increased CR participation [33, 39], reinforcing the longstanding importance of physician referral [50]. Patients often reported seeking medical reassurance before enrolling in CR and were more likely to attend when guided by a trusted provider. This finding indicated the importance of patient support, particularly robust referral pathways that position CR as a standard component of secondary prevention rather than an optional add-on in their recovery. Automated referral systems have become popular for streamlining enrolment, but this method cannot mimic the influence of personal endorsement from primary care physicians or cardiologists [15], whose recommendations carry significant weight in patients’ decision-making [51].

Programme delivery design shaped patient engagement. For instance, face-to-face models were associated with higher completion rates than phone-based approaches [15]. Meanwhile, telehealth CR was perceived as lacking adequate mental health support, supervised exercise training, and opportunities for peer interaction, despite reducing barriers related to distance, travel, cost, and scheduling [52]. These outcomes suggested that optimising CR delivery requires tailoring programme components to the needs of specific patient subgroups instead of relying on uniform, one-size-fits-all models, besides ensuring that professional endorsement and programme structure support long-term engagement effectively.

Logistical challenges significantly hampered CR participation among patients, particularly geographic distance, transportation difficulties, and travel time. Notable regional variation was observed within the same country, as reported in Malaysia (27–70%) [35, 37] and China (5–25%) [25, 30]. The Malaysian population is distributed across Peninsular Malaysia and East Malaysia. Issues such as living far from CR centres or lacking access to private transportation were more pronounced in East Malaysia, making patients less likely to participate in CR programmes [37]. Similar patterns exist in China, where substantial geographic diversity, a large rural population, and the concentration of CR services in urban tertiary hospitals create significant access barriers. Patients were often frustrated with restricted and paid hospital parking [25], compounding existing travel and logistical difficulties and contributing to low participation.

Accessible medical care is commonly defined as being within a 30-minute travel radius [53], but studies examining journey-time thresholds for CR discovered that travel periods exceeding 60 minutes significantly reduced the likelihood of patient referral and participation [37, 54]. Findings from broader Asian settings suggested that the relationship between transport mode and participation is context-dependent. While self-driving facilitates attendance in some regions [37], non-drivers in urban areas with reliable public transportation may be more likely to participate in CR [35] due to reduced parking constraints and easier access to hospital facilities. This outcome was consistent with reports from Malaysian public hospitals, where long parking waits and limited capacity create additional barriers for patients who rely on private vehicles [55].

Work- and time-related conflicts remain major barriers to CR participation, with non-participants frequently citing inflexible working hours, job demands, and scheduling constraints as reasons for non-attendance [11, 25, 27, 28]. These challenges underscore the need for flexible programme formats, including home-based, hybrid, and after-hours options to accommodate working adults. Comorbidities exert an additional influence on participation and adherence, as chronic conditions such as diabetes, chronic obstructive pulmonary disease, and other cardiovascular risk factors were consistently associated with reduced involvement or higher dropout rates [11, 12, 15, 38]. Interestingly, findings from Chong *et al*. [35] diverge from this pattern, reporting higher participation among patients with multiple comorbidities in a mobile-based CR programme. This discrepancy may reflect the role of delivery modality, indicating the potential of flexible, technology-enabled models in reducing physical and logistical burdens experienced by multimorbid patients. Taken together, personalised and flexible CR designs that address occupational demands and the complexity of patients’ health profiles are superior in promoting CR participation compared to uniform, centre-based models.

Participation in CR within multiracial settings, such as Malaysia, demonstrated notable ethnic disparities, with Chinese and Indian patients participating at substantially lower rates than their Malay counterparts [35, 37]. These gaps extend beyond demographic proportions, highlighting broader socioeconomic inequalities, differences in health literacy, cultural perceptions of CR, and unequal access to healthcare services [56]. Financial barriers further exacerbated these disparities and consistently influence CR uptake across regions. Underprivileged groups face limited access due to programme costs [25, 27], and European findings associated lower income with significantly lower participation [57, 58]. In China, the absence of CR reimbursement under the National Health Service restricted affordability for outpatient follow-up, contributing to low engagement [25].

Lower socioeconomic status and education levels impeded CR participation more broadly [29, 31, 33], highlighting the need for subsidised programmes and targeted educational interventions to improve awareness and access. Employment status adds to the complexity of CR participation. While employed individuals often report time constraints, some evidence suggests that unemployed individuals may be more willing to participate due to greater schedule flexibility [12]. Collectively, these findings demonstrated that socioeconomic inequities, financial barriers, and differing life circumstances were critical in shaping CR participation, underscoring the need for context-responsive strategies that promote equitable access across diverse populations.

Age is a consistent determinant of CR participation and adherence, with middle-aged adults engaging at higher rates than older individuals [29, 31]. Older adults, regardless of gender, were less likely to enrol in or sustain structured exercise programmes [59]. Meanwhile, the oldest cohorts, particularly octogenarians, remain markedly underrepresented in CR research and programme design despite their substantial potential to benefit [60]. Several factors contributed to reduced engagement among older adults, including misconceptions about the impact of CR in health improvement, greater emotional distress, social isolation, and socioeconomic or functional limitations [61]. In contrast, younger patients tend to be more proactive in managing risk factors and perceive CR as valuable for long-term health. The consistent decline in participation with increasing age underscores the need to adapt CR delivery models, such as through hybrid or telehealth options, tailored support, or caregiver involvement, to ensure that programmes are accessible, acceptable, and responsive to the needs of older adults.

Evidence on gender influence on CR participation remains inconclusive. Some studies report higher engagement among men [12, 31, 37], whereas others report greater participation among women [29]. These conflicting patterns may reflect contextual differences. For example, women face additional practical and sociocultural barriers that limit attendance in several Asian settings, including caregiving responsibilities, transportation, comorbidities, and family obligations [37, 62]. In contrast, increasing public awareness and gender-focused cardiovascular campaigns in parts of Europe may have enhanced women’s engagement with CR [29].

Contrary to the relatively larger number of included studies examining gender, only a small subset explored mental health, also reporting inconsistent findings in this area. Depression has been associated with non-completion and reduced participation in CR [15, 63] and empirical evidence suggested that emotional distress arising from long waiting times and limited peer support may further discourage engagement [15]. Conversely, patients with better mental health management demonstrated higher completion rates, particularly in home-based CR models [38]. Meanwhile, some studies reported that anxiety increased the likelihood of participation [35, 64], while others demonstrated a negative relationship [65]. Collectively, these inconsistencies—combined with the limited number of mental health–focused studies—indicated that the effects of gender and psychological factors on CR uptake were highly context-dependent and shaped by sociocultural, emotional, and health-system influences, highlighting the need for more nuanced, stratified research and tailored intervention strategies.

Social support consistently emerged as a strong facilitator of CR participation and adherence. Patients who received encouragement from family members, friends, or community networks were more likely to enrol in and complete CR [12, 33, 35], whereas those who lived alone or lacked social support showed higher rates of dropout or non-completion [15, 36]. Notably, family and peer endorsement can shape patients’ perceptions of CR and reinforce decisions to engage in rehabilitation [66]. Evidence from Zhang *et al*. [24] further illustrated the value of structured social support, demonstrating that participants involved in the “Life Club” initiative were significantly more likely to complete CR than those without such support. These findings underscore the need for formal or informal social support integration in CR programmes to strengthen motivation, accountability, and sustained engagement among participants.

This scoping review has several strengths and limitations. A key strength of this study was the inclusion of evidence from industrialised and developing countries, enabling a comprehensive understanding of barriers across diverse healthcare and socioeconomic contexts. Furthermore, this study addressed a gap in earlier studies on mental health as an underevaluated yet potentially important factor influencing CR engagement, highlighting the need for further research to clarify the role in CR. In addition, only studies reporting a minimum of 100 participants were included in this review to reduce small-sample bias and improve evidence robustness. Nonetheless, several limitations should be acknowledged. Some data that were utilised in this study lack quantitative indicators, such as standard errors, measures of variance, correlation coefficients, and effect sizes, which precluded the possibility of conducting a meta-analysis. A systematic review with sufficient homogeneous data would allow for pooled estimates and provide stronger global evidence to enhance understanding of barriers to CR utilisation.

## 5. Conclusion

Participation and adherence to CR remain suboptimal globally despite the proven benefits. This scoping review demonstrated that barriers to engagement were multifactorial and varied across contexts. Demographic and psychosocial challenges were predominant in industrialised settings, whereas developing regions face pronounced financial, geographical, and infrastructural limitations. Meanwhile, issues such as transportation, comorbidities, and work demands affect patients universally. Engagement is further shaped by patient perceptions, physician endorsement, programme design, and the availability of social and mental health support, yet evidence regarding gender and psychological influences remains limited and inconsistent. Addressing these disparities will require tailored, context-responsive approaches that strengthen individual support, incorporate flexible delivery models such as hybrid or telehealth CR, and implement policy-level measures to reduce financial and logistical barriers. These strategies are essential for improving equitable access and optimising the impact of CR worldwide.

## Supplementary Material

Supplementary material associated with this article can be found, in the online version, at https://doi.org/10.31083/RCM45898.
